# Research on Prediction of Preterm Birth Risk Based on Digital Twin Technology

**DOI:** 10.3390/diagnostics16030499

**Published:** 2026-02-06

**Authors:** Xinyuan Chen, Renyi Hua, Yanping Lin

**Affiliations:** 1 School of Mechanical Engineering, Shanghai Jiao Tong University, Shanghai 200240, China; xinyuan_123@sjtu.edu.cn; 2 International Peace Maternity and Child Health Hospital, Shanghai Jiao Tong University School of Medicine, Shanghai 200030, China; renyi3551_cn@me.com

**Keywords:** digital twin, personalized healthcare, preterm birth risk prediction, machine learning, clinical decision support

## Abstract

**Background:** Preterm birth remains a major cause of perinatal morbidity and long-term developmental complications. Existing prediction methods often lack individualized assessment and have limited capability to integrate multi-source maternal–fetal information. This study aims to develop a personalized preterm birth risk prediction model and to construct a visual, interactive digital twin platform that enhances clinical communication and supports early risk identification. **Methods:** A total of 1157 structured clinical records collected from 2020 to 2024 were preprocessed through automated feature typing, missing-value handling, and normalization. Two complementary machine-learning models—FT-Transformer and Light Gradient Boosting Machine (LightGBM)—were trained and calibrated to produce probabilities. Their outputs were fused using a Stacking Logistic Regression framework to improve prediction stability and calibration. A 3D visualization module was developed using 3ds Max, PyQt6, and PyVista to generate personalized uterine–fetal models based on fetal position, placental location, and Biparietal Diameter (BPD), enabling synchronized display of prediction results. **Results:** The fused model achieved an AUC of 0.820, PR-AUC of 0.405, a Brier score of 0.040, and an expected calibration error (ECE) of 3.39 × 10^−3^, demonstrating superior discrimination and probability reliability compared with single models. The interactive platform supports real-time data input, risk prediction, and adaptive 3D rendering, providing clear and intuitive visual feedback for clinical interpretation. **Conclusions:** The integration of machine learning fusion and digital twin visualization enables individualized assessment of preterm birth risk. The system improves model accuracy, enhances interpretability, and offers a practical tool for clinical follow-up, risk counseling, and maternal health education.

## 1. Introduction

Preterm birth, defined as delivery before 37 completed weeks of gestation, remains a leading cause of perinatal mortality and long-term morbidity worldwide. It is strongly associated with neonatal death, neurodevelopmental impairment, and increased risks of chronic cardiovascular and metabolic disorders later in life. Given its complex, multifactorial etiology, extensive obstetric research has focused on identifying clinical predictors and developing surveillance strategies to assess early risk.

Current medical approaches to preterm birth prediction primarily rely on maternal history, cervical length measurement, biochemical screening, and uterine artery Doppler indices. Although these indicators are clinically interpretable and widely used, their predictive performance is often limited when applied in isolation, and their effectiveness varies substantially across gestational stages and patient populations [1,2]. Moreover, many risk assessment strategies are conducted at discrete time points and are predominantly informative in mid- to late pregnancy, thereby limiting opportunities for timely intervention. Inter-individual variability in maternal physiology, placental development, and pregnancy trajectories further complicates individualized risk stratification, particularly in low-prevalence clinical settings [3].

Consequently, the development of individualized preterm birth prediction models within a digital twin-oriented research paradigm has emerged as an important research direction at the intersection of medicine and engineering, particularly in obstetrics and gynecology [4,5].

More broadly, recent advances in artificial intelligence have demonstrated substantial potential across diverse medical domains. In medical imaging, Esteva et al. showed that deep neural networks can achieve dermatologist-level performance in skin cancer classification using clinical images [6]. For clinical risk prediction, Rajkomar et al. applied deep learning models to large-scale electronic health records to predict inpatient mortality and adverse outcomes, highlighting their ability to capture complex temporal patterns [7]. In cardiovascular medicine, Attia et al. developed an artificial intelligence-based electrocardiogram algorithm for early detection of atrial fibrillation during sinus rhythm [8]. In oncology, Topol reviewed the role of artificial intelligence in improving diagnostic accuracy and advancing personalized cancer care [9]. More recently, Johnson et al. discussed integrating machine learning models into clinical workflows, emphasizing reliability, interpretability, and generalizability as key challenges for real-world deployment [10].

Digital twin technology, which combines virtual simulation, artificial intelligence, and big data, holds broad promise across various medical fields. It enables the development of personalized care, surgical planning, disease prediction, medication management, and support for research and clinical trials [11]. For personalized medical plans, Elisa Tardini et al. developed a digital twin system (DQL) for head and neck cancer. It uses patient data to optimize decisions about survival rates and chemotherapy side effects [12]. In surgical planning, Aubert’s team created digital twin models for patients with tibial fractures using image segmentation. They evaluated the risk of secondary fractures under different fixation methods. This helps select a surgical plan [13]. For disease prediction, Lal et al. built digital twins for critically ill patients. These twins predict the response of sepsis patients to treatment and help adjust treatment plans in a timely manner [14]. For individualized medication management, Bahrami’s team used pharmacokinetic/pharmacodynamic models. They simulated the blood concentration and analgesic effect of fentanyl patches. This approach aims to optimize pain management [15]. Digital twins also aid in the design and optimization of medical devices. For example, they simulate valve performance using a patient’s digital heart model. This helps improve diagnosis and surgical planning [16].

By leveraging recent advances in artificial intelligence and data-driven modeling, digital twin-oriented frameworks enable individualized, multi-parameter risk modeling and enhanced visualization for preterm birth, facilitating earlier risk identification and informed clinical decision-making. It should be noted that, under current clinical and ethical constraints in obstetric research, such frameworks do not constitute fully realized digital twins in the strict engineering sense, but rather represent practical, data-driven approximations toward this paradigm. Accordingly, this study aims to develop a preterm birth prediction framework that integrates multi-dimensional clinical measurements with a digital modeling and visualization approach, together with a three-dimensional representation of the uterus and fetus, to provide clinicians with a personalized tool for early risk assessment and timely intervention.

## 2. Materials and Methods

### 2.1. Data Source and Processing

This study utilized clinical data obtained from the International Peace Maternity and Child Health Hospital of the China Welfare Institute. After institutional ethical approval and de-identification, a total of 1157 pregnancy records collected between 2020 and 2024 were included for analysis, comprising 51 preterm birth cases and 1106 term birth cases. The clinical features were primarily collected during the mid-second trimester (approximately 18–24 weeks of gestation), while preterm birth was defined as delivery before 37 completed weeks of gestation.

Prior to model development, the raw clinical data were organized into a structured tabular format and underwent systematic preprocessing. An automated type inference mechanism was first applied based on each variable’s data type and the number of distinct values, following standard preprocessing practices in machine learning-based clinical prediction tasks. An initial set of 43 candidate features was classified into numerical and categorical variables [17,18]. Numerical variables included quantitative measurements such as placental growth factor concentration, crown–rump length, and mean arterial pressure, whereas categorical variables represented discrete clinical attributes such as smoking status, diabetes type, and previous delivery method. Integer-valued features with limited distinct categories were treated as categorical variables, while continuously distributed features were defined as numerical variables, providing a consistent basis for subsequent preprocessing.

Missing values in numerical variables were imputed using mean substitution, while missing entries in categorical variables were assigned a dedicated “Missing” category to enhance reproducibility. These strategies represent widely adopted baseline approaches for handling missing data in medical machine learning, balancing simplicity, stability, and sample retention [19]. To improve data stability and reduce multicollinearity, features with excessive missing rates or strong linear redundancy were excluded. For example, fetal weight was excluded because of its strong deterministic relationship with biometric parameters, such as biparietal diameter. After this filtering process, 35 clinically relevant features were retained as model inputs. No automated feature selection algorithms were applied; instead, feature inclusion was guided by data quality considerations and established clinical knowledge.

Finally, all numerical features were normalized to the [0, 1] range to mitigate scale-related effects during model training. Feature normalization is a standard preprocessing step that improves numerical stability and ensures comparability across heterogeneous features and learning algorithms [20].

### 2.2. Construction of the Digital Model

LightGBM and FT-Transformer were selected for their complementary strengths in structured clinical data modeling. LightGBM has been widely adopted for medical risk prediction due to its robustness, computational efficiency, and strong performance with heterogeneous tabular features, particularly in settings with limited sample sizes and class imbalance. In contrast, FT-Transformer is specifically designed for tabular data and leverages attention mechanisms to flexibly model high-order feature interactions, making it well-suited for capturing complex relationships among clinical variables.

During model design, other commonly used approaches, including logistic regression, random forests, and multilayer perceptrons, were also considered. However, prior studies have demonstrated that gradient boosting-based tree models consistently achieve superior performance in clinical risk prediction using structured health records, often outperforming traditional statistical models [21,22]. More recently, transformer-based architectures tailored for tabular data have shown competitive or even superior performance by effectively modeling complex feature interactions, thereby providing a powerful complement to tree-based methods [23]. Therefore, LightGBM and FT-Transformer were chosen as representative and complementary base learners in the proposed framework.

#### 2.2.1. Selection and Application of the FT-Transformer Model

The FT-Transformer is a deep learning model designed for tabular data. It converts each column of features into vectors. It then establishes connections between features using a self-attention mechanism. The model excels at capturing non-linear and high-order interaction relationships. It performs well, demonstrates multiple benchmarks, and is regarded as a significant deep tabular model in recent years [23]. Deep learning models, such as the FT-Transformer, offer distinct advantages over tree-based models. They can automatically learn complex interactions and identify cross-column combinatorial relationships. However, FT-Transformer models show poor stability and weak resistance to noise when handling small or medium-sized datasets. They also struggle with data containing a high number of features [24]. In this study, the FT-Transformer serves as a deep baseline model to capture complex nonlinear relationships among clinical variables. To improve prediction stability on smaller datasets, it was combined with a tree-based model. This compensates for shortcomings under such data scale conditions.

During the model training and validation phase, this study used cross-entropy (a measure of the difference between predicted and actual values) as the primary loss function for the binary classification task. To address the difficulty of identifying minority-class samples (such as cases of “preterm birth”), a class weight mechanism—a method to give more importance to rare cases—was introduced into the loss function. This enhanced attention to the minority class. An early stopping strategy (where training stops when performance stops improving) was employed during the training process. The Precision-Recall Area Under the Curve (PR-AUC), a performance metric for imbalanced data, was used as the monitoring metric to prevent overfitting on the validation set. Finally, the model weights that showed the most stable performance on the validation set were retained for subsequent testing and deployment.

#### 2.2.2. Introduction to the LightGBM Model

LightGBM is an efficient implementation of gradient boosting decision trees (GBDT), a machine learning technique used for regression and classification. It employs a leaf-wise growth strategy, which splits the most promising leaf first. LightGBM also employs feature bundling, which combines rarely used features to reduce dimensionality, as well as other techniques. These contribute to training efficiency, noise resistance, and practicality. LightGBM is particularly suitable for modeling small and medium-sized clinical tabular data, which often contain missing values and discrete features. It demonstrates stable predictive performance [25]. Compared to deep learning models, LightGBM generalizes well for mixed-type data in small to medium-sized sample scenarios. It also features relatively simple hyperparameter tuning and robust model output. However, it is less flexible than deep learning models in capturing implicit high-order interaction relationships between features.

In this study, we selected LightGBM as the tree-based baseline model. The core goal is to fully learn strong feature signals in tabular data and generate stable, reliable sample ranking and probability estimation. To further improve model performance and prediction stability, this paper integrates LightGBM with FT-Transformer. This combination is designed to leverage both models for complementary enhancement.

During the model training phase, this study addressed class imbalance by assigning appropriate weights to samples of both the positive and negative classes. Key hyperparameters, such as tree depth, number of leaf nodes, and learning rate, were constrained to prioritize model stability. During validation, PR-AUC was used as the indicator for early stopping. Before finalizing the model, predicted probabilities were calibrated to better match the actual risk distribution [26].

#### 2.2.3. Multi-Model Fusion Strategy

During the multi-model fusion phase, LightGBM and FT-Transformer are treated as heterogeneous base learners that independently generate calibrated probability outputs. Three integration strategies—Probability Averaging (Blend-Prob), Log-Odds Averaging (Blend-Logit), and Stacked Logistic Regression (Stacking-LR)—are systematically compared to examine their implementation processes, applicable contexts, and performance implications within the proposed framework [27].

Probability Averaging (Blend-Prob): This method calculates the weighted average of output probabilities from the FT-Transformer and LightGBM, with optimal weights determined through a grid search. It is straightforward to implement, offering low deployment costs, and is preferred when base models have similar performance, limited complementarity, or when reproducibility and maintenance are prioritized.

Log-Odds Averaging (Blend-Logit): This method converts the output probabilities of FT-Transformer and LightGBM into log-odds, then calculates their weighted fusion. The approach helps handle extreme values and can alleviate overfitting to extreme samples in class-imbalanced tasks [28].

Stacked Logistic Regression (Stacking-LR): This method compares the outputs of FT-Transformer and LightGBM by using them as feature inputs in a multi-layer ensemble learning framework. A Logistic Regression classifier, trained on the out-of-fold (OOF) probabilities from both base models, serves as the secondary classifier to combine their complementary strengths and produce the final classification [29,30,31].

**Figure 1:**
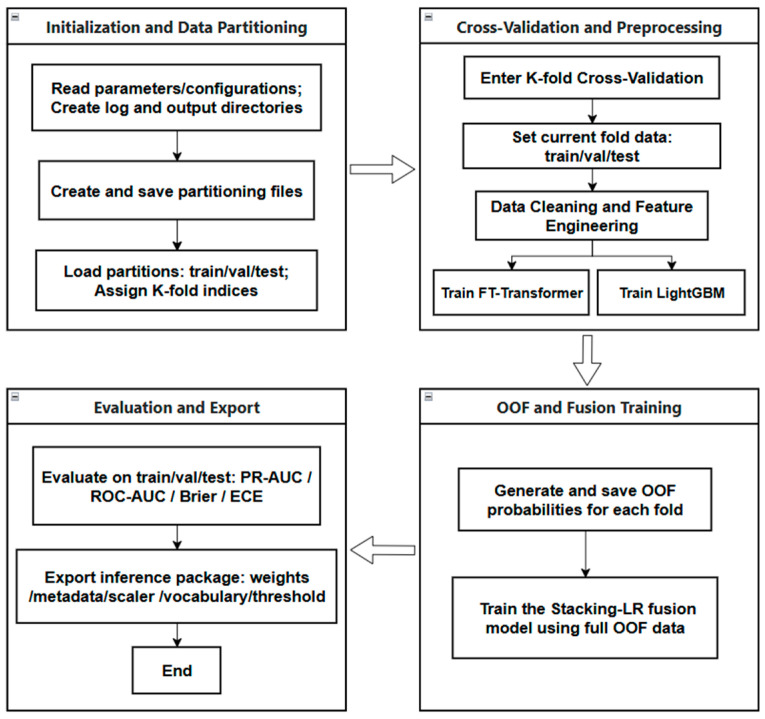
Overall workflow of the proposed preterm birth prediction framework, including data partitioning, K-fold cross-validation, out-of-fold probability generation, multi-model fusion, and performance evaluation.

The proposed preterm birth prediction framework first applies strict data partitioning to define the training, validation, and independent test sets. Subsequently, 5-fold cross-validation is performed within the training data to train base models and generate out-of-fold prediction probabilities. These out-of-fold probabilities are then used to construct a stacking-based fusion model. Finally, multiple performance metrics are used to systematically evaluate the model, and the trained models are exported for subsequent inference and deployment. As illustrated in Figure 1, this workflow is designed to prevent information leakage and ensure robust and reproducible model evaluation.

### 2.3. Construction of 3D Models

**Figure 2:**
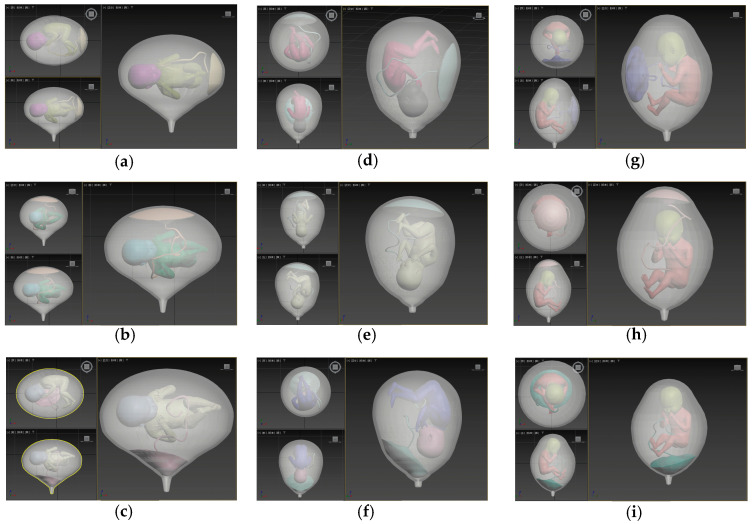
Representative configurations of the three-dimensional uterus–fetus models. Subfigures (**a**–**i**) illustrate nine discrete anatomical configurations defined by combinations of fetal presentation and placental attachment: (**a**) transverse presentation with anterior placenta; (**b**) transverse presentation with fundal placenta; (**c**) transverse presentation with placenta previa; (**d**) cephalic presentation with anterior placenta; (**e**) cephalic presentation with fundal placenta; (**f**) cephalic presentation with placenta previa; (**g**) breech presentation with anterior placenta; (**h**) breech presentation with fundal placenta; (**i**) breech presentation with placenta previa. Color variations are used solely for visual differentiation of modeling components during 3D construction and do not encode quantitative, physiological, or predictive information.

This study constructed a three-dimensional (3D) fetal visualization model using Autodesk 3ds Max, implementing nine discrete anatomical configurations defined by all combinations of fetal presentation (cephalic, breech, transverse) and placental attachment (anterior, fundal, previa), as illustrated in Figure 2. In clinical practice, placental attachment also includes posterior placenta. However, in the proposed modeling framework, posterior attachment was not parameterized separately because, in the absence of an explicit maternal anatomical reference, anterior and posterior placental locations are geometrically and visually symmetric within the uterus–fetus–placenta system. Therefore, the posterior placenta was subsumed into the anterior category as a modeling abstraction. The 3D model incorporates a parameterized adjustment framework for fetal head geometry, enabling user-driven scaling and rotation based on direct clinical metrics such as Biparietal Diameter (BPD). Algorithmically managed NURBS-based surface blending ensures anatomically contiguous skin meshes at the head–body junction, supporting morphometric consistency for individualized risk stratification.

It should be noted that the three-dimensional uterine–fetal models in this study do not directly participate in the predictive computation nor provide feedback to the model parameters or structure. The prediction model and the 3D visualization module are computationally decoupled, sharing only selected maternal–fetal features as input. Predicted risk probabilities are used solely to synchronize and drive the visualization outputs.

#### 2.3.1. Modeling of the Uterus and Fetus

For individualized 3D anatomical visualization, this study implemented a procedurally generated, highly parameterized model encompassing the uterus, fetus, and placenta. The uterine structure is represented as a thin-walled volumetric mesh with variable wall thickness and ellipsoidal aspect ratios, parametrically defined by a dual-surface B-spline formulation. Placental geometry utilizes a parametric NURBS surface mesh algorithm, supporting three canonical attachment patterns—anterior, fundal, and previa—each mapped via adaptive mesh conformation to the inner uterine (endometrial) surface using non-rigid mesh fitting algorithms. The umbilical cord is modeled as a spline-driven cylindrical mesh with endpoints rigidly constrained to the placental disk and fetal umbilicus, and is capable of algorithmic curvature adjustment to align with clinically observed anatomical variation.

The fetal model simulates three clinically significant orientations: cephalic, transverse, and breech positions. To preserve morphometric integrity during head scaling and repositioning, we introduce an algorithmic protocol for cranio-cervical segmentation and reconnection. This involves extracting a consistent anatomical reference ring from the fetal trunk mesh, which serves as a reference plane for the precise segmentation of the cranial structure. Surface continuity across the head-trunk interface is achieved through a computationally generated quadrilateral mesh that bridges the strips. Sequential rigid alignments are performed using the extracted ring centroid, while proportional head scaling is dictated by the numerical BPD input. Post-scaling, a compensatory translation vector is computed to restore the original spatial relationship between the segmented cranial and trunk structures.

The system dynamically selects and executes geometric asset generation based on the active clinical input parameters, including fetal orientation, placental attachment, and BPD. This process enables the automatic construction and rendering of the uterus, placenta, and fetus within a unified 3D scene graph. The resultant meshes serve as anatomically precise carriers for the context-specific visualization of predictive risk indices, thereby facilitating the integration of high-fidelity diagnostic workflows.

#### 2.3.2. Model Recognition and Calling Mechanism

Automated model instantiation and management are coordinated by a backend scheduling algorithm that ingests fetal orientation, placental attachment type, and BPD as input parameters. The algorithm retrieves and instantiates the relevant 3D geometric resources. When data fields are incomplete, predefined system defaults are programmatically applied. Head mesh scaling is executed via a function of the BPD input, while spatial correction employs centroid alignment algorithms and translation of the reference neck ring. System-generated bridging strips and algorithmic mesh suturing maintain continuous topology at segmented boundaries. Post-generation quality assurance encompasses automated checks for mesh integrity and visual coherence, conducted prior to GPU-based scene rendering, as well as synchronous communication with the risk prediction output module. Manual user intervention is systematically excluded at all stages of the process, regardless of parameter heterogeneity.

### 2.4. System Integration and Software Design

This study developed a preterm birth prediction platform that integrates predictive algorithms and 3D visualization, utilizing the Python 3.10.18 and PyQt6 frameworks. In the process flow, the system’s backend receives user input data, processes this data with the prediction algorithm, and immediately returns the results to the user interface. Updated outputs are reflected in the visualization module, forming a continuous closed-loop workflow. The platform features a modular architecture that supports functional expansion, multi-user access, and data encryption, meeting clinical deployment requirements.

### 2.5. Setting of Model Evaluation Metrics

To ensure a fair and robust assessment of model generalization while preventing information leakage, the dataset was first partitioned into a training/validation set (80%) and an independent test set (20%) using stratified sampling. The test set was strictly held out throughout model development and used exclusively for final performance evaluation. Within the training/validation set only, 5-fold stratified cross-validation was applied during model training: base models were trained on four subsets and generated predictions for the remaining subset. This process yielded out-of-fold (OOF) probability estimates for all training samples, providing an unbiased characterization of model behavior on unseen data and forming the basis for evaluation-stage error analysis, probability calibration, and stacking-based fusion via logistic regression.

Given the low prevalence of preterm birth (approximately 4.4%), multiple complementary metrics were adopted to comprehensively evaluate model performance. Discriminative ability was assessed using the area under the receiver operating characteristic curve (AUC) and precision–recall AUC (PR-AUC), while probabilistic accuracy and calibration quality were evaluated using the Brier score and Expected Calibration Error (ECE). The final AUROC values and ROC curves were computed solely on the independent test set using the predicted probabilities from the finalized model, ensuring an unbiased estimate of clinical predictive performance.

## 3. Results

### 3.1. Results of Model Performance Metrics

In this study, we systematically evaluated the performance of various single models and fusion strategies for preterm birth prediction in terms of discriminative ability, probabilistic accuracy, and calibration effectiveness. The evaluation metrics included the area under the receiver operating characteristic curve (AUC), the precision–recall AUC (PR-AUC), the Brier score, and the Expected Calibration Error (ECE). The comparative results of different models under these metrics are illustrated in Table 1.

Among single models, FT-Calib demonstrated good discriminative performance (AUC = 0.801, PR-AUC = 0.403); however, its relatively higher Brier score (0.041) and ECE (1.39 × 10^−4^) indicated suboptimal probability calibration. In contrast, LGBM-Calib exhibited slightly lower discriminative ability (AUC = 0.809, PR-AUC = 0.326) but achieved superior calibration performance (Brier = 0.040, ECE = 4.9 × 10^−5^), making it more suitable for applications requiring accurate probability estimation. The weighted fusion models (Blend-Prob and Blend-Logit) howed discriminative performance comparable to FT-Calib but did not yield substantial improvements in calibration.

Overall, the Stacking-LR fusion model achieved the best performance across all evaluation metrics. It achieved an AUROC of 0.820 (95% CI: 0.659–0.945) and a PR-AUC of 0.405, indicating strong discriminative capability. It should be noted that PR-AUC values are highly sensitive to class prevalence; given the low prevalence of preterm birth in this dataset, moderate PR-AUC values are expected and remain informative for ranking high-risk cases. Meanwhile, AUC provides a prevalence-independent measure of global discrimination, and calibration-oriented metrics such as the Brier score and ECE offer complementary insight into the reliability of predicted probabilities. The Stacking-LR model achieved a low Brier score (0.040) and stable calibration performance, suggesting that the observed performance gains reflect not only improved discrimination but also enhanced probabilistic accuracy.

### 3.2. Demonstration of the Visualization System

**Figure 3:**
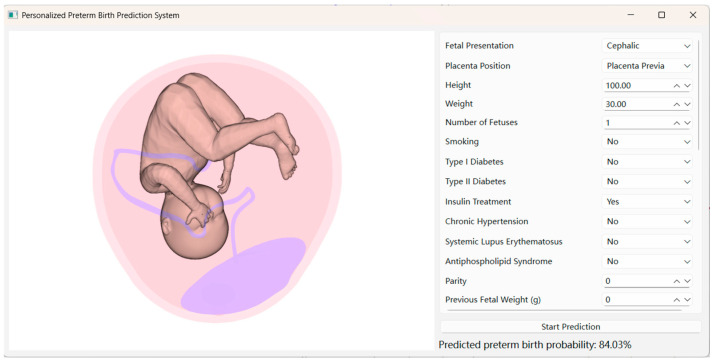
Visualization interface demonstrating the linkage between individualized three-dimensional uterine–fetal models and predicted preterm birth risk probability.

The interface of this system integrates 3D visualization and display of prediction results to enhance user experience and interpretability. The three-dimensional models serve solely as a visualization layer, synchronized with the predicted risk outputs, and do not participate in the prediction process. As shown in Figure 3, the left panel utilizes the PyVista library to render three-dimensional models of the fetus, uterus, placenta, and umbilical cord in real time, with model dimensions dynamically adjusted according to input fetal characteristics, such as head circumference and body length, ensuring consistency between the visual representation and the corresponding clinical measurements. The right panel contains a structured input form that allows users to enter standardized information, including basic demographics, medical history, and fetal characteristics. After the user submits their inputs by clicking “Start Prediction,” the backend first validates and preprocesses the input features, then invokes the trained prediction model to perform inference.

The resulting preterm birth risk probability is subsequently returned to the interface and displayed numerically, while the same output is used to synchronize the corresponding three-dimensional visualization. This step-wise execution ensures consistent data flow between input, prediction, and visualization, integrating data entry, risk computation, and 3D presentation within a unified workflow. 

Specifically, the stacking-based fusion model is invoked after feature preprocessing to generate the final preterm birth risk probability, which is then displayed in the interface and used to synchronize the corresponding three-dimensional visualization.

## 4. Discussion

### 4.1. Interpretation of Model Fusion Performance in the Context of Previous Studies

The Stacking-LR fusion model achieved the strongest overall performance across both discrimination and calibration metrics. Its superior AUC and PR-AUC indicate that combining diverse learners can effectively enhance minority-class detection in imbalanced medical datasets. This finding is consistent with recent ensemble-learning studies showing that stacked generalization improves robustness and reduces overfitting in clinical prediction tasks involving varied feature distributions [32]. Additionally, the fusion model’s ability to sustain good calibration supports evidence that hierarchical blending of deep and tree-based models increases the reliability of probabilities in high-stakes healthcare prediction scenarios [33].

The FT-Transformer component contributed to capturing complex non-linear interactions, a recognized advantage of attention-based architectures for tabular medical data [34]. In parallel, the LightGBM component enhanced calibration stability, consistent with findings that gradient boosting is especially effective for low-prevalence clinical outcomes [35]. Collectively, these strengths validate our hypothesis that model complementarity can meaningfully improve estimates of preterm birth risk.

### 4.2. Clinical Interpretation of Predicted Risk Scores

The proposed model’s output represents an individualized probability of preterm birth rather than a definitive diagnosis. From a clinical perspective, lower predicted risk values may support routine surveillance, whereas elevated risk scores indicate increased susceptibility and may prompt closer monitoring, additional examinations, or early preventive interventions. Importantly, the absolute magnitude of the predicted probability should be interpreted in the context of population prevalence and individual clinical background, and is primarily intended for risk stratification rather than threshold-based decision-making. Therefore, the proposed framework serves as a decision-support tool to assist clinicians in prioritizing follow-up intensity and counseling, while final clinical decisions remain under physician judgment.

### 4.3. Comparison with Existing Preterm Birth Prediction Research

Traditional biomarkers, such as fetal fibronectin (fFN), offer only short-term predictive windows, limiting their usefulness for early intervention [36]. By contrast, this study provides second-trimester prediction capability, allowing clinicians to take preventive action well before symptoms emerge. This extended window supports research advocating for earlier identification of mechanical and biochemical pathways associated with preterm birth [37].

Most machine-learning studies on preterm birth have focused on structured clinical variables and rarely integrate individualized medical history, lifestyle factors, or anatomical variation into a unified framework [38]. The digital twin approach adopted here represents a methodological advancement by embedding personalized morphology and risk factors into the model. This aligns with recommendations for next-generation preterm birth prediction tools that emphasize multimodal integration and individualized thresholds.

### 4.4. Limitations and Future Directions

Despite the promising results, several limitations must be acknowledged regarding both methodological scope and external validity. Under the practical constraints of obstetric research, the proposed framework does not represent a fully realized digital twin system in the strict engineering sense. Rather, it serves as a digital twin-oriented predictive and visualization framework aimed at supporting individualized risk assessment.

The internal validity of this study is reinforced by a rigorous experimental design, which includes strict separation of an independent test set, five-fold cross-validation within the training data, and out-of-fold probability estimation to prevent information leakage and ensure unbiased performance evaluation. Employing complementary discrimination- and calibration-oriented metrics further strengthens the robustness of the reported findings.

Nevertheless, limitations affecting external validity persist. First, this study draws on data from a single medical center, and the relatively low prevalence of preterm birth in the dataset may limit sensitivity for detecting the minority class—a well-known challenge in obstetric risk prediction research [3]. Future research will prioritize expanding to multi-center datasets and conducting external validation to enhance generalizability. Second, the current framework uses parameterized three-dimensional anatomical templates that do not fully capture patient-specific imaging variability. Integrating reconstructions based on ultrasound, MRI, or generative models may improve anatomical fidelity and clinical relevance. Third, while probability calibration is acceptable, further refinement is required to ensure stable deployment amid potential distributional shifts. Incorporating time-series modeling, dynamic risk trajectories, and continuous physiological signals represents a key direction for advancing clinically applicable digital twin-oriented systems. Collectively, these efforts will strengthen the robustness and clinical utility of the proposed framework.

## Figures and Tables

**Table 1:** Comparative performance of single models and fusion strategies for preterm birth prediction, evaluated using discrimination and calibration metrics on the independent test set.

Model	PR-AUC	AUC	Brier	ECE
FT-Calib	0.403	0.801	0.041	1.39 × 10^−4^
LGBM-Calib	0.326	0.809	0.040	4.9 × 10^−5^
Blend-Prob	0.404	0.815	0.041	6.8 × 10^−5^
Blend-Logit	0.405	0.817	0.041	3.0 × 10^−5^
Stacking-LR	0.405	0.820	0.040	3.39 × 10^−3^
Adaptive-Conf	0.402	0.800	0.041	8.2 × 10^−5^

## Data Availability

The clinical data used in this study were obtained from Shanghai International Peace Maternity & Child Health Hospital under IRB approval (details above). Due to patient privacy and institutional policy, the de-identified individual-level dataset cannot be made publicly available. De-identified data may be shared upon reasonable request to the corresponding author and with prior approval from the hospital’s Ethics Committee/IRB and a signed Data Use Agreement. The data are preserved on the hospital’s secure server for at least five years after publication.

## Abbreviations

The following abbreviations are used in this manuscript:

AUC	Area Under the Receiver Operating Characteristic Curve
BPD	Biparietal Diameter
ECE	Expected Calibration Error
fFN	Fetal Fibronectin
LGBM/LightGBM	Light Gradient Boosting Machine
PR-AUC	Precision–Recall Area Under the Curve
FT-Transformer	Feature Tokenizer Transformer
Stacking-LR	Stacking Logistic Regression
PyQt6	Python Qt Framework Version 6
PyVista	Python VTK-based 3D Visualization Package
3ds Max	Autodesk 3ds Max
